# Ketone ester ingestion increases exogenous carbohydrate storage and lowers glycemia during post-exercise recovery: a randomised crossover trial

**DOI:** 10.1007/s00394-025-03784-w

**Published:** 2025-08-12

**Authors:** Lizzie Clarke, Sophie L. Russell, Bruno Spellanzon, Jennifer L. Maher, James A. Betts, Javier T. Gonzalez

**Affiliations:** 1 Centre for Nutrition, Exercise and Metabolism, University of Bath, Bath, UK; 2 Department for Health, University of Bath, Bath, BA2 7AY UK

**Keywords:** Ketones, Sports nutrition, Glucose metabolism, Exercise, Endurance

## Abstract

β-hydroxybutyrate can suppress endogenous glucose production, with potential implications for carbohydrate metabolism during post-exercise recovery. The aim of the current study was to assess the effects of ketone ester ingestion during post-exercise recovery, on carbohydrate metabolism and subsequent exercise capacity. Thirteen endurance-trained men (age: 18–61 years, maximal aerobic capacity: 50 to 73 mL kg^−1^ min^−1^) completed two conditions in a randomized crossover design. During both conditions, participants performed two exhaustive bouts of running separated by 4 h of recovery, during which they ingested sucrose (1 g kg^−1^ h^−1^ and high natural abundance ^13^C) and whey protein (0.4 g kg^−1^ h^−1^) beverages. In one condition, the beverage was supplemented with 0.29 g kg^−1^ h^−1^ of ketone monoester (KETONE), in the other, the beverage was supplemented with an isoenergetic (fat), taste-matched placebo (PLACEBO). Breath samples were analysed for CO_2_ production and ^13^C enrichment to determine the fate of ingested carbohydrate. Blood was sampled to examine metabolite and insulin concentrations. KETONE increased blood β-hydroxybutyrate concentrations (> 3.5 mmol L^−1^ versus PLACEBO, *p* < 0.0001) and retention of ingested sucrose (from 206 ± 26 g with PLACEBO to 220 ± 26 g with KETONE, *p* = 0.001) while lowering glycemia (> 1 mmol L^−1^ versus PLACEBO, *p* < 0.0001). This occurred with no evidence of increased gastrointestinal distress during recovery, but mild additional lower gastrointestinal distress during the second run (*p* = 0.03). There was no evidence for differences in time-to-exhaustion during the second run (PLACEBO:54 ± 33 min, KETONE:52 ± 28 min; *p* = 0.87). In conclusion, ketone ester ingestion during post-exercise recovery augments retention of ingested carbohydrates and lowers glycemia. No evidence for increased exercise capacity was detected during subsequent running.

**Supplementary Information:**

The online version contains supplementary material available at 10.1007/s00394-025-03784-w.

## Introduction

Carbohydrates in the form of glycogen and glucose are key fuels for endurance exercise performance [1–3]. Consequently, prolonged bouts of exercise deplete whole-body carbohydrate stores and changes in post-exercise metabolism contribute to the restoration of carbohydrate stores and thereby achieve homeostasis [3–5].

Several nutritional interventions can further accelerate the restoration of depleted carbohydrate stores, with the primary focus being the ingestion of sufficient amounts of carbohydrates. Ingesting 1 to 1.2 g per kilogram body mass per hour of carbohydrates robustly stimulates net muscle glycogen synthesis, with protein co-ingestion providing added benefit for muscle glycogen synthesis when carbohydrate intake is suboptimal (i.e. ~0.8 g per kilogram body mass per hour) [6]. Ingestion of glucose-fructose mixtures (e.g., sucrose) can further increase liver glycogen restoration and subsequent exercise capacity [7–10]. Therefore, glucose-fructose mixtures with added protein provide a solid initial foundation for post-exercise glycogen recovery strategies to build upon.

With events or training schedules requiring several bouts of exercise within 24 h (e.g. stage racing in cycling, multiday ultraendurance running, and tournament style competition), additional strategies to further accelerate post-exercise recovery are warranted. Ketone bodies produce several physiological effects that could influence post-exercise carbohydrate metabolism and thereby accelerate post-exercise recovery. β-hydroxybutyrate infusion to produce circulating concentrations ~ 0.5 mmol L^−1^ suppresses endogenous glucose production [11], which may explain the observations that ingesting ketone esters during post-exercise recovery lowers glycemia [12]. β-hydroxybutyrate can also be utilized by skeletal muscle and brain, thereby sparing utilization of other fuels [11]. In a postprandial state, suppression of endogenous glucose production and/or utilization of ketone bodies as substrates may result in greater storage of ingested carbohydrates that could contribute to subsequent exercise performance [7, 9, 10, 13]. Whilst some studies have investigated net skeletal muscle glycogen synthesis with ketone esters during post-exercise recovery [14, 15], the focus on skeletal muscle alone would miss any potential effects on liver glycogen metabolism that contribute to whole-body carbohydrate storage. Whether ingesting ketone esters during recovery from exhaustive exercise do, in fact, increase retention of ingested carbohydrates, is currently unknown. Accordingly, the aim of the present study was to assess the effects of ketone ester ingestion during recovery from exercise on carbohydrate metabolism (glycemia and retention of ingested carbohydrates) and on subsequent exercise capacity.

## Methods

### Study design

This study was a single-blind, randomized crossover design with 2 experimental conditions separated by at least a 7-day washout between visits (max 6 weeks; median 14 days). The protocol received favorable opinion from the University of Bath, Biomedical Sciences Research Ethics Committee (5202-6834) and was registered at clinicaltrials.gov (NCT06846840). Participants provided informed, written consent prior to participation. The study was conducted in line with the latest version of the Declaration of Helsinki [16].

### **Participants**

Participants were endurance-trained adult males from the area local to Bath (recruited and tested between November 2024 and February 2025) and were aged 18–65 years, with a peak oxygen uptake (V̇O_2_peak) of at least 45 mL kg^−1^ min^−1^, at least 2 years running experience and at least 3 h of endurance training per week. Exclusion criteria included a body mass index of > 30 kg m^−1^, being a habitual smoker within the last 5 years, history of uncontrollable metabolic or respiratory disease, currently taking medication, following a low-carbohydrate diet or consuming ketone supplements, or a history of irritable bowel syndrome. All exclusion criteria were based on self-reporting.

### Randomization

Following screening and eligibility assessment, participants completed a preliminary test to determine maximal aerobic capacity and the running velocity for the main laboratory visits. Following this, participants were allocated to the randomly generated sequence of conditions which used unrestricted randomization using www.randomizer.org by JG (Supplementary Table 1).

### Preliminary tests

Preliminary testing to determine participant height, body mass, and aerobic capacity took place one week prior to the first main laboratory visit. Following measurement of body mass (Seca 803 Clara Digital Scale, Seca GmbH., Hamburg, Germany) and height (Stadiometer, Holtain Limited., Pembrokshire, UK), participants completed a two-part exercise test on a motorized treadmill (Woodway LG70, Woodway GmbH., Meil am Rhein, Germany). Part-one consisted of an incremental sub-maximal exercise test to ascertain oxygen uptake relative to running speed. Participants completed 4 × 4 min stages, starting at self-selected warm up speed and increasing by 1 km h^−1^ each four min. In the final minute of each stage, heart rate (Polar RS400, Polar, Kempele, Finland), ratings of perceived exertion (RPE; Borg, 1973), and expired breath samples (Hans Rudolph Douglas Bag Systems, Cranlea Human Performance Limited, UK) were collected.

Following a rest period of ~ 10 min, an incremental incline test to volitional exhaustion was performed to determine V̇O_2_peak. Speed was held constant at a pace equivalent to their final stage in part one, while incline was increased by 1% each minute to task failure. Participants were instructed to signal the point at which they felt they could perform one further minute of exercise, at which a one-minute expired breath sample was collected, and heart rate was recorded. The speeds eliciting 50%, 70%, and 90% V̇O_2_peak were then determined using simple regression analyses for use in subsequent experimental trials.

### Pre-visit standardization

All participants were permitted to continue their habitual training throughout the study period but refrain from caffeine on the morning of visits and from alcohol during the 48 h prior to main laboratory visits. On the morning of main laboratory visits, participants were advised to consume their habitual breakfast and attempt to energy match this between trials (confirmed via text message). During all stages of testing, participants were permitted to drink water *ad libitum* while total fluid intake was recorded.

### Main laboratory visits

Upon arrival at the laboratory (between 0800 and 0830), participants rested for 10-minutes and then gas exchange was determined using a 5-minute Douglas bag sample. Following this, participants completed two exhaustive bouts of running separated by a 4-hour recovery period. Running bout 1 started with a 5-minute warm up (50% VO_2_peak), followed by 2-minute intervals alternating between 90% VO_2_peak and 50% VO_2_peak. When 90% VO_2_peak was no longer sustainable, the intensity was reduced to 80% VO_2_peak and then 70% VO_2_peak as per prior work [7, 8]. The point at which 70% VO_2_peak could no longer be sustained marked the end of running bout 1 and the beginning of the recovery period. After the 4-hour recovery period, participants ran to exhaustion at 70% VO_2_peak.

During recovery, participants ingested recovery beverages every 30 min which contained carbohydrate (sucrose at 1.0 g kg^−1^ hr^−1^; Tate and Lyle Pure Cane Granulated Sugar) and protein (whey protein hydrolysate, 0.4 g kg^−1^ hr^−1^; MyProtein Vanilla Whey). Participants then ingested either ketone monoester (R)-3-hydroxybutyrate (R)-3-hydroxybutyl (KETONE, 0.29 g kg^−1^ hr^−1^; DG, TDS Ltd., Oxford, UK), or a taste and energy matched placebo (PLACEBO, olive oil with 0.5 µL mL^−1^ of Bitrex^®^ bitter flavor; Bitrex Solution B, Alpha Solway, UK). The original protocol plan was to use medium-chain triglycerides as the placebo as reported on the clinical trials registry. However, before the study began, the decision was taken to use olive oil in place of medium-chain triglycerides as olive oil has less potential to induce ketosis. Fat was selected over carbohydrate or protein to energy-match the placebo being the least influential on whole-body carbohydrate metabolism. The cane sugar was obtained in 5 batches and the ^13^C enrichment was determined for each batch for later calculations [mean ± SD enrichment: − 2.12 ± 0.19‰ versus pee dee belemnite (PDB)]. During recovery, fingertip capillary blood samples were also taken before ingestion of the first beverage, and every 30 min thereafter. Further fingertip capillary samples were taken at 15 min into running bout 2, and then at the point of exhaustion. A 12-mL exetainer vial, and 5-minute Douglas bag samples of exhaled breath were also taken before, and every 60 min after ingestion of the first recovery beverage. Gastrointestinal symptom questionnaires were administered at the end of recovery and the end of running bout 2.

### Blood sampling and analysis

At each timepoint, fingertip capillary blood samples were collected in 70-µL heparinized capillary tubes (Radiometer, Denmark) and 300-µL EDTA microvettes (Sastedt, Germany). Blood β-hydroxybutyrate concentrations were determined using a hand-held monitor (Optium Neo, Abbott, USA). Capillary tubes were analysed for blood glucose, lactate, pH and bicarbonate concentrations using a blood gas analyzer (ABL90 Flex Plus, Radiometer, Denmark). The microvettes were centrifuged at 9503 × *g* for 10 min at room temperature (Hareus Biofuge Pico; DJB Labcare). The extracted plasma samples were aliquoted into 0.5-mL tubes (model 5810; Eppendorf) and stored in a freezer at − 70 °C before later analysis. Plasma insulin concentrations were analyzed in singular using a solid-phase 2-site enzyme immunoassay (Mercodia Insulin ELISA; Mercodia). All samples from each participant were run on the same ELISA plate. Within-plate CV was < 3%.

### Breath sampling and analysis

Breath samples were collected using the Douglas bag method to establish rates of oxygen consumption and carbon dioxide production. Five-minute samples were taken after 5-min equilibration periods. Concurrently, ambient O_2_ and CO_2_ concentrations were measured to account for changes in inspired gas concentrations [17]. Concentrations of O_2_ and CO_2_ were measured in a known volume of sample (Mini MP 5200, Servomex Ltd.), and the total volume of expired gas was determined by evacuation using a dry gas meter (Harvard Apparatus). To determine ^13^C enrichment of expired CO_2_, breath samples were collected in 12-ml exetainers (Labco Ltd.), filled in duplicate by 10-s exhalation. Whole-body substrate oxidation was calculated from V̇O_2_ and V̇CO_2_ according to stochiometric equations (Frayn, 1983; Jeukendrup & Wallis, 2005). The ^13^C/^12^C ratio of expired CO_2_ was determined by continuous flow isotope ratio mass (Isolab Scientific Ltd., UK) and the enrichment expressed as δ per mil difference between the ^13^C/^12^C ratio of the sample and a known standard. Exogenous sucrose oxidation rates were then calculated according to the following equation.

$$ {\text{Exogenous}}\;{\text{sucrose}}\;{\text{oxidation}} = \dot{V}CO_{2} \times ~\frac{{\left( {\delta {\text{Exp}} - ~\delta {\text{Exp}}_{{{\text{bkg}}}} } \right)}}{{\left( {\delta {\text{Ing}} - ~\delta {\text{Exp}}_{{{\text{bkg}}}} } \right)}}\left( {\frac{1}{{k1~ \times ~k2}}} \right) $$

Where exogenous sucrose oxidation rates are in g min^−1^. V̇CO_2_ is in L min^−1^. δExp is the breath CO_2_ enrichment at a given time point. δExp_bkg_ is the breath CO_2_ enrichment prior to beverage ingestion. δIng is the enrichment of the beverage, and *k*1 and *k*2 are the L of V̇CO_2_ generated by oxidation of 1 g of carbohydrate (0.7467 L) and the fractional recovery of ^13^C on breath (0.54) [18], respectively. Sucrose retention was calculated as total ingested sucrose minus exogenous sucrose oxidation.

### Urine sampling and analysis

Participants were asked to void upon arrival at the laboratory, and all urine from then on during the laboratory visit was collected. The total volume was recorded, and a 1-mL aliquot was stored at − 70 °C for later analysis of urea concentrations using an automated spectrophotometer (RX Daytona+, Randox, Ireland). Prior to each assay, the analyzer was calibrated using a zero (0.9% w/v NaCl) and a commercially available standard and checked against high- and low-quality controls (Randox, Ireland). Within-run precision was confirmed when assayed quality controls were within an acceptable range of expected values. The coefficients of variation across runs was ≤ 3%. Urine urea concentrations (mmol L^−1^) were converted into total urinary urea output over the 4 h of recovery by multiplying urinary urea concentration by volume of urine output, and then multiplying by 0.18 to convert from mmol to g.

### Gastrointestinal symptom questionnaire and blinding interview

Gastrointestinal symptoms were assessed using a previously published questionnaire containing 4 questions for each category of upper gastrointestinal symptoms (heartburn, bloating, nausea and vomiting), lower gastrointestinal symptoms (intestinal cramps, abdominal pain, flatulence and diarrhea), and systemic symptoms (dizziness, headache, muscle cramp, urge to urinate) [19]. Each question was rated on a scale of 0 to 8 (0 = none, 1–2 = mild, 3–4 = moderate, 5–6 = severe, 7–8 = unbearable). Success of blinding was assessed using an exit interview where participants were asked: (1) whether they could identify a difference between the beverages; and (2) to attempt to identify which beverage was the placebo and which was the experimental beverage.

### Sample size justification

Whilst the primary aim of the current study was to assess the effect of ketone ester ingestion on ingested carbohydrate retention, the primary outcome to form the basis of the sample size justification was based on the reduction in glucose concentrations that we previously observed with ketone ester ingestion during recovery from exercise [12]. The rationale for this was due to an absence of data on the expected effect size on ingested carbohydrate retention and reductions in glucose concentrations being related to the metabolism of ingested carbohydrates to provide an appropriate outcome measure to base the sample size justification on. In our previous study, we found the ketone ester ingestion lowered glucose concentrations by 0.75 ± 0.59 mmol L^−1^ compared with control. Using this effect size (*d* = 1.27), 9 participants would provide > 90% power to detect an effect size of this magnitude with a two-tailed test and an alpha level of 0.05 in a crossover design. To account for dropout, we aimed to recruit at least 15 participants.

### Statistical analyses

Data were analysed in Prism v10.2.3 (GraphPad Software, Boston, MA). Distribution of data was assessed by visual inspection of Q-Q plots. Across all data there were 5% missing values (103 out of 2003 data points). Where any of these were between two other data points during the recovery period, the mean of the data points from either side of the missing data was taken. For time-series data, when no data were missing a two-way (time x condition) repeated measures ANOVA was employed. Where missing data remained, a mixed effects model was employed. For summary data or specific timepoints (total oxidation, urine output etc.), two-sided, paired Student’s *t*-tests were performed to assess differences between conditions.

## Results

Eighteen participants were recruited and five dropped out prior to completing the study due to injury (*n* = 3), lack of time (*n* = 1) or lack of tolerance to the test drinks (*n* = 1). Therefore, thirteen participants completed the study (Table 1). One participant developed an injury during their PLACEBO time-to-exhaustion test and so *n* = 12 for data relating to running bout 2.

**Table 1:** Participant characteristics

	Mean ± SD	Range
Age (y)	29 ± 12	18 to 61
Height (cm)	178 ± 6	167 to 187
Body mass (kg)	69 ± 7	58 to 80
Body mass index (kg m^−1^)	21.9 ± 2.1	18.3 to 25.4
Maximal aerobic capacity (mL kg^−1^ min^−1^)	62 ± 8	50 to 73
Endurance training experience (years)	5 ± 2	2 to 10

*n* = 13 endurance trained males

The distance covered during running bout 1 was 12.9 ± 1.8 km during PLACEBO, and 13.0 ± 1.9 km during KETONE. Time taken to reach exhaustion during running bout 1 was 49.8 ± 1.5 min during PLACEBO, and 50.2 ± 1.6 min during KETONE.

Eight (62%) participants identified a difference between the intervention drinks, one (8%) recalled this as a taste and viscosity difference. When asked to identify the order of their experimental condition, five (38%) correctly guessed their intervention condition, and eight (62%) incorreclty guessed their intervention condition.

**Fig. 1:**
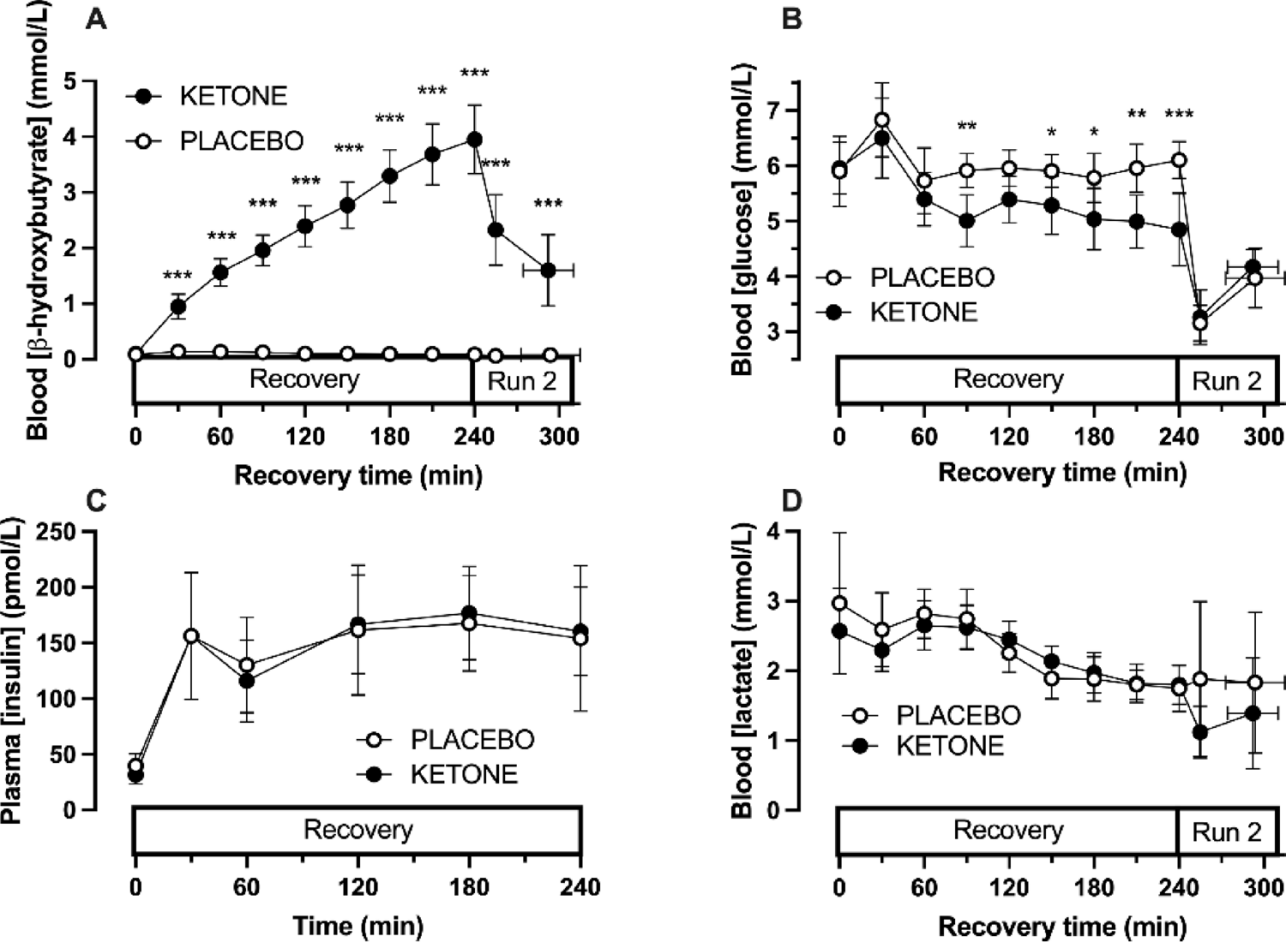
Circulating β-hydroxybutyrate (**A**) glucose (**B**) insulin (**C**) and lactate (**D**) concentrations during recovery from prolonged running with ingestion of sucrose and whey protein with either placebo (PLACEBO) or ketone ester (KETONE), and during a second bout of running to exhaustion (Run 2). Data are means ± 95%CI. *n* = 13 endurance-trained men. *p < 0.05, **p < 0.01, ***p < 0.001, KETONE vs. PLACEBO

During the recovery period, blood β-hydroxybutyrate concentrations remained below the limit of detection during PLACEBO, but rose to ~ 4 mmol L^−1^ during KETONE and remained elevated compared with PLACEBO through recovery and the second running bout (condition x time interaction, *p* < 0.0001, Fig. 1A). In contrast, over the course of the recovery period blood glucose concentrations were lowered by KETONE, compared with PLACEBO with a difference of > 1.2 mmol L^−1^ by the end of the recovery period (condition x time interaction, *p* = 0.007, Fig. 1B), but there was no evidence of differences between conditions in blood glucose concentrations (*p* = 0.64 and *p* = 0.57 at minute 15 and exhaustion, respectively). Plasma insulin concentrations rose during the recovery period (time effect, *p* < 0.0001), with no evidence of differences between conditions (condition effect, *p* = 0.89; condition x time interaction, *p* = 0.69, Fig. 1C). Blood lactate concentrations fell during the recovery period (time effect, *p* < 0.0001), with no evidence of differences between conditions during recovery or during running bout 2 (condition effect, *p* = 0.62; condition x time interaction, *p* = 0.07, *p* = 0.24 and *p* = 0.35 at minute 15 and exhaustion, respectively; Fig. 1D).

**Fig. 2:**
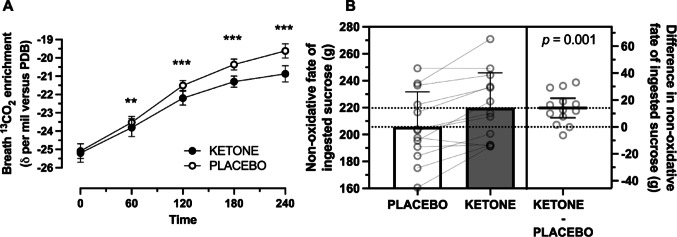
Breath ^13^C enrichment of CO_2_ (**A**) and ingested sucrose retention (**B**) during recovery from prolonged running with ingestion of sucrose and whey protein with either placebo (PLACEBO) or ketone ester (KETONE). Data are means ± 95%CI. *n* = 13 endurance-trained men. *p < 0.05, **p < 0.01, ***p < 0.001, KETONE vs. PLACEBO. PBD, pee dee belemnite

Breath ^13^C enrichment of CO_2_ increased during the recovery period (*p* < 0.0001), to a greater extent in PLACEBO compared with KETONE (*p* < 0.0001, Fig. 2A), which translated into more exogenous sucrose retention with KETONE compared with PLACEBO (*p* = 0.001, Fig. 2B).

**Fig. 3:**
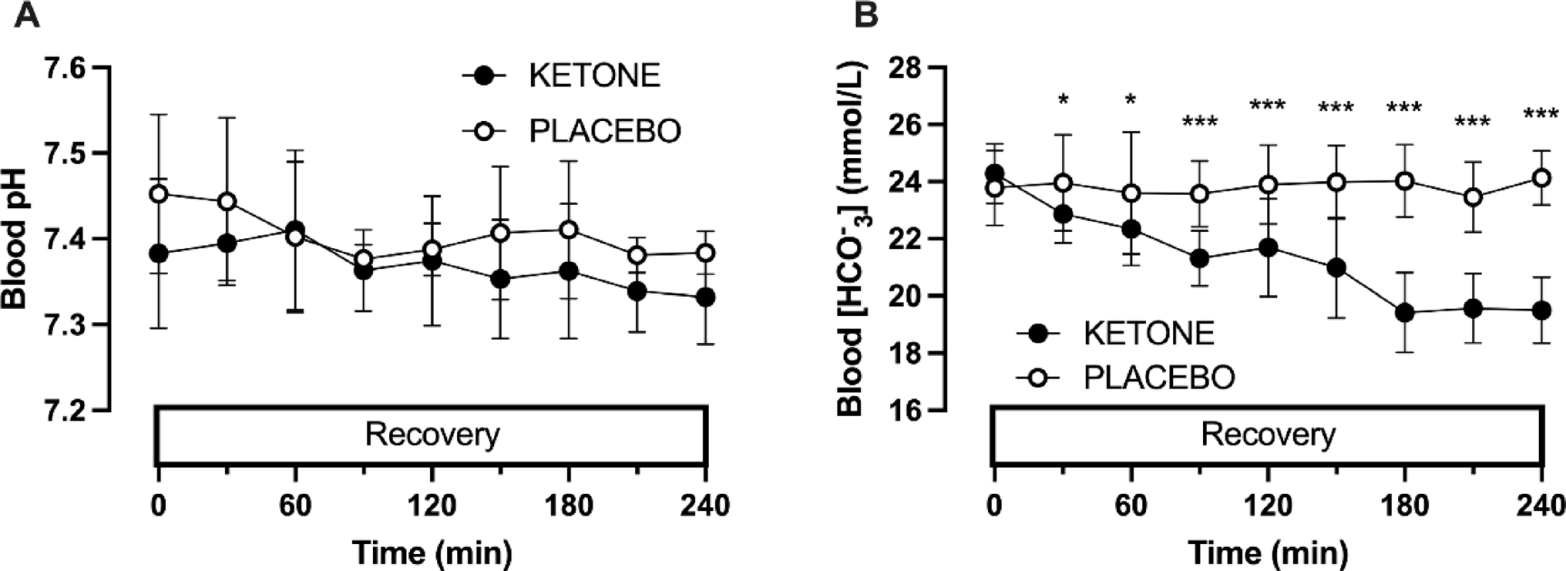
Blood pH (**A**) and bicarbonate concentrations (**B**) during recovery from prolonged running with ingestion of sucrose and whey protein with either placebo (PLACEBO) or ketone ester (KETONE). Data are means ± 95%CI. *n* = 13 endurance-trained men. *p < 0.05, **p < 0.01, ***p < 0.001, KETONE vs. PLACEBO

There was no evidence that blood pH changed over time (time effect, *p* = 0.14), or that blood pH was altered by KETONE ingestion *versus* PLACEBO (condition effect, *p* = 0.10; condition x time interaction, *p* = 0.73, Fig. 3A). In contrast, HCO^−^ _3_ concentrations decreased during recovery with KETONE versus PLACEBO (condition x time interaction, *p* < 0.0001; Fig. 3B).

Voluntary fluid intake and urine output were both lower with KETONE compared with PLACEBO (Table 2). However, there was no evidence of a difference between conditions in either urine urea concentrations nor total urine urea output (Table 2).

**Table 2:** Fluid intake, urine and Urea output during post-exercise recovery while ingesting carbohydrate and protein beverages with placebo (PLACEBO) or isoenergetic ketone ester (KETONE)

	PLACEBO	KETONE	*p* value
Fluid intake (mL)	3116 ± 538	2835 ± 455	0.01
Urine output (mL)	1359 ± 410	1186 ± 499	0.02
Urine [urea] (mmol L^− 1^)	95 ± 9	108 ± 38	0.13
Urine urea excretion (g 4 h^− 1^)	5.7 ± 1.5	5.3 ± 1.6	0.33

Data are means ± SD, *n* = 13.

**Fig. 4:**
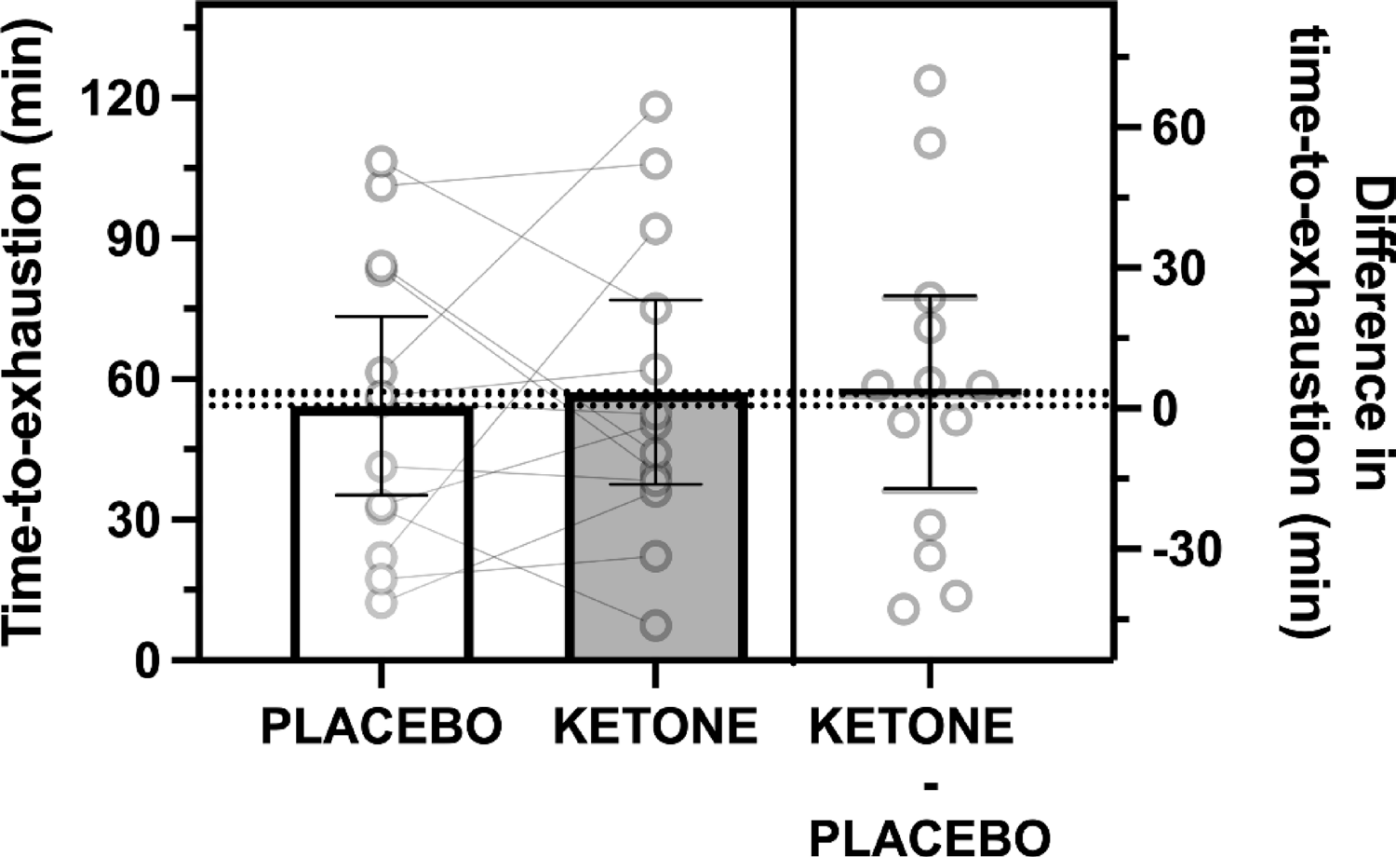
Time-to-exhaustion during a second bout of running after recovery from prior prolonged running with ingestion of sucrose and whey protein with either placebo (PLACEBO) or ketone ester (KETONE). Data are means ± 95%CI. *n* = 12 endurance-trained men

There was no evidence of differences between conditions in time-to-exhaustion (*p* = 0.87, Fig. 4).

**Fig. 5:**
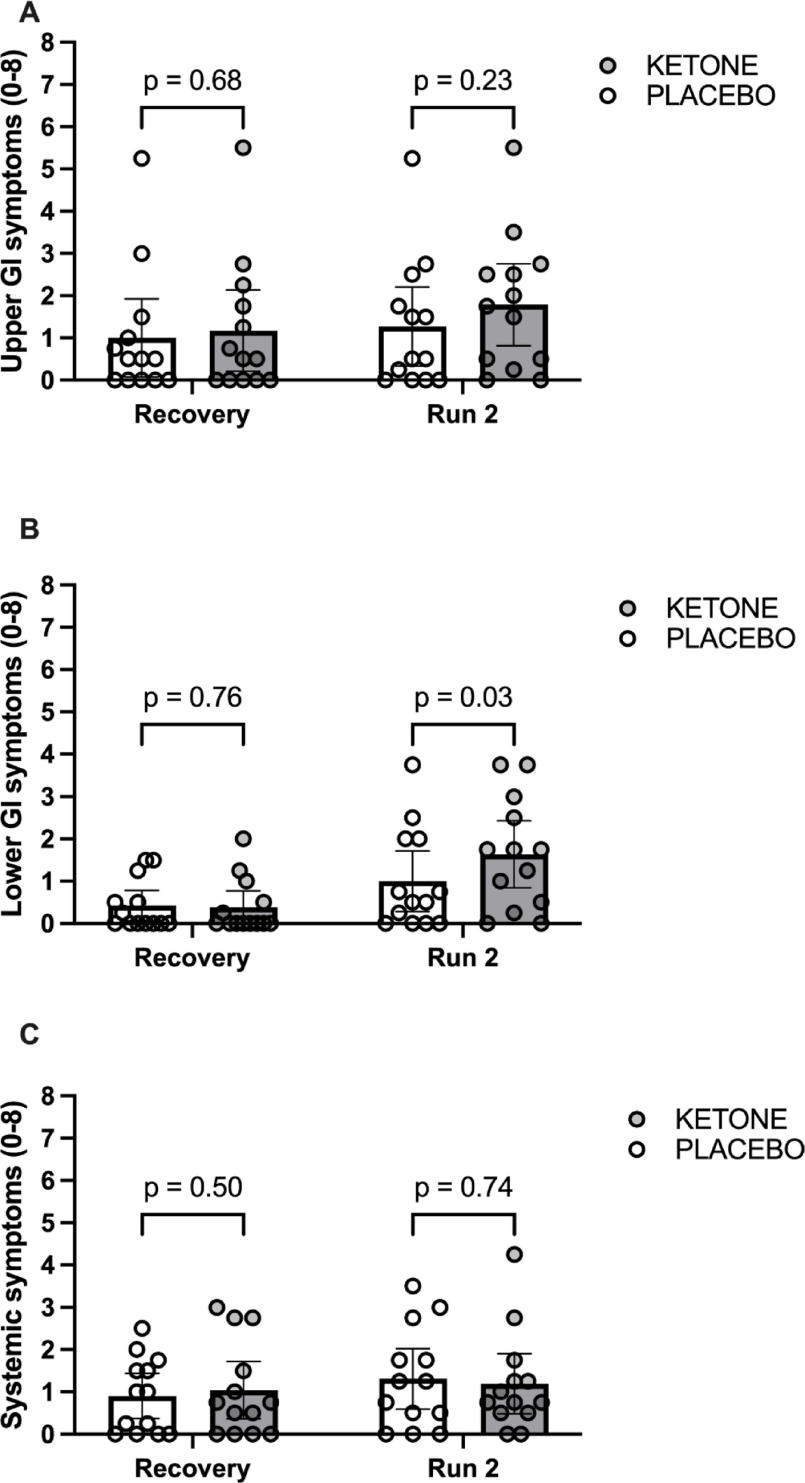
Upper gastrointestinal (**A**; GI), lower GI (**B**), and systemic symptoms (**C**) during recovery from prior prolonged running with ingestion of sucrose and whey protein with either placebo (PLACEBO) or ketone ester (KETONE) and during a subsequent second bout of running. Data are means ± 95% CI. *n* = 13 endurance-trained men

Gastrointestinal symptoms were all mild during the recovery period, with no evidence for differences between conditions (Fig. 5A, B and C). During running bout 2, lower gastrointestinal symptoms were higher with KETONE *versus* PLACEBO (*p* = 0.02, Fig. 5B), albeit still reported as “mild” on average and no evidence was observed for differences between conditions in upper gastrointestinal symptoms or systemic symptoms (Fig. 5A and C).

## Discussion

The present study assessed the effects of ketone ester ingestion on post-exercise metabolism and subsequent exercise capacity. Ketone ester ingestion increased retention of ingested carbohydrates and lowered glycemia, with no evidence for an improvement in subsequent exercise capacity. Ketone ester ingestion also lowered plasma bicarbonate concentrations without any meaningful or statistically significant differences in lactate or insulin concentrations.

Increased retention of ingested carbohydrates could reflect temporary storage in the stomach (delayed gastric emptying) and/or storage as glycogen in the liver or skeletal muscle (or as lipid via *de novo* lipogenesis) [20]. Slower gastric emptying would slow systemic glucose appearance rates, lower insulinemia and may increase upper gastrointestinal symptoms such as bloating. We examined ratings of gastrointestinal symptoms and whilst we did observe some mild lower abdominal symptoms which were higher following ketone ester ingestion compared with placebo, there was no evidence for increased upper gastrointestinal symptoms with ketone ester *versus* placebo ingestion. Therefore, subjective ratings of gastrointestinal discomfort suggest that slower gastric emptying is unlikely to be the primary underlying mechanism for increased ingested carbohydrate retention in the current study. Furthermore, reduced intestinal absorption rates would result in lower insulinemia from reduced exogenous glucose appearance rates [21], and lower muscle glycogen resynthesis rates, yet we observed little difference in insulinemia with ketone ester *versus* placebo ingestion and others report either similar or higher muscle glycogen resynthesis with ketone ester ingestion during post-exercise recovery [14, 15]. Finally, when gastric emptying has been assessed using the acetaminophen test, there is no evidence that ingesting ketone esters delays gastric emptying rates [22]. Therefore, the data in the current study combined with prior evidence, indicate that increased exogenous sucrose retention is unlikely to be explained by reduced gastric emptying rates, leaving glycogen synthesis as a potential fate of the retained carbohydrate.

Previous studies assessing the effects of ketone ester ingestion on glycogen metabolism during post-exercise recovery have reported conflicting findings, with some demonstrating an increase [14], and others reporting no evidence of an increase in net muscle glycogen synthesis [15]. It is possible that this can be (partly) explained by differences in carbohydrate provision, with increased net muscle glycogen synthesis seen with hyperglycemic clamp, but not with oral carbohydrate ingestion. Intravenous glucose infusion prioritises the muscle as a major site of glucose disposal. Oral carbohydrate ingestion, however, results in splanchnic exposure to carbohydrate prior to peripheral deliver to muscle and results in more emphasis on splanchnic extraction. This emphasises the importance of the liver for postprandial carbohydrate metabolism and suggests that increased exogenous sucrose retention observed in the present study is likely due to increased glycogen storage in liver rather than skeletal muscle. This is also supported by plausible mechanisms by which ketone esters could accelerate liver glycogen storage. Raising circulating β-hydroxybutyrate to 0.5 mmol L^−1^ can suppress endogenous glucose production by ~ 20%, independent from insulinemia [11]. Decreasing endogenous glucose production would result in greater retention of ingested carbohydrates in the liver as glycogen, and would also explain the current observed reduction in glycemia in the presence of similar insulinemia, which is also supported by work in type 2 diabetes [23]. Taken together, this suggests that ketone esters may result in increased net liver glycogen storage, and the current study provides the justification to invest in future studies to confirm (or refute) this.

Increased retention of ingested carbohydrates during recovery may have implications for subsequent exercise metabolism and performance. If this retention reflects net glycogen storage, then this can provide additional carbohydrate-based fuel for subsequent exercise, contributing to maintenance of carbohydrate availability and glycemia. However, we did not find evidence for differences in exercise capacity, glycemia or markers of glycolytic flux (lactate concentrations) during subsequent exercise with ketone ester versus placebo ingestion. It is possible that the exercise test was not limited by glycemia and carbohydrate availability, which is supported by the lack of observed hypoglycemia at the end of exercise. It is also possible that the increased lower gastrointestinal symptom burden with ketone esters contributed to offsetting any metabolic advantage for exercise capacity. Further work is required to establish the potential for ketone esters to affect subsequent exercise capacity with modalities and intensities of exercise where liver glycogen and hypoglycemia may be limiting.

The principle of using ^13^C enriched carbohydrates to probe metabolic handling following ingestion is subject to several assumptions that warrant discussion within the context of this study. The primary assumption is that the ^13^C appearance on breath CO_2_ fully reflects metabolism of the ingested carbohydrate [24]. One theoretical challenge to this assumption is that protein and/or ketone body accumulation and oxidation alter CO_2_ production by acid-base disturbance and oxidation [25]. A second theoretical challenge to this assumption is “trapping” of ^13^C in the bicarbonate pool or tricarboxylic acid (TCA) cycle. We found little difference in urine urea excretion between conditions which suggests protein oxidation was unlikely to meaningfully differ with ketone ester *versus* placebo ingestion. At rest, exogenous β-hydroxybutyrate oxidation has been shown to be relatively limited in vivo, at ~ 0.012 g min^−1^ [26]. This would translate to ~ 0.01 L min^−1^ and thus reflects a negligible contribution (< 3%) to CO_2_ production [25]. Each mol of β-hydroxybutyrate is thought to reduce CO_2_ by ~ 1 mol from acid-base disturbances. Based on the blood β-hydroxybutyrate concentrations we observed, this disturbance would also only affect CO_2_ production by < 0.01 L min^−1^ (< 2%) [25]. For trapping of ^13^C in the bicarbonate pool or TCA cycle, the slightly reduced blood bicarbonate concentration we observed with ketone ester versus placebo suggests a reduced bicarbonate pool size which would, if anything, result in an underestimate of ingested sucrose retention. Aside from the above discussion, the challenges to the assumption for stable isotope tracers are primarily relevant to quantitative values, and relative differences between conditions are still likely to be valid, since the way the challenges to assumptions would violate relative differences would mainly be if they disproportionally influenced ^13^C *versus* ^12^C. The fundamental assumption of stable isotope tracer methods is that the chemical similarity of the tracer and tracee means that the tracer behaves biochemically identically to the tracee [24] and there is no reason to expect that the above considerations would disproportionally affect ^13^C over ^12^C. Therefore, these theoretical challenges are unlikely to practically influence the main interpretation of our data.

The current study was conducted in male athletes for pragmatic reasons of trial scheduling within the data collection timeframe for the project. This may limit the generalisability to female athletes and therefore future research may investigate the influence of ketone ester ingestion in females on carbohydrate metabolism in recovery, in addition to whether there are sex-based differences in the responses to ketone ester ingestion. A further limitation with the current study is the lack of generalisability of the recovery timeframe to many competitions such as stage racing in road cycling, where the recovery timeframe is typically over 4 h. Nevertheless, there are scenarios where this timeframe is relevant. For example, in most ultramarathons rest periods and sleep is on the race clock and thus athletes choose short breaks of a few hours or less. A further rationale for this timeframe was based on prior work where differences in liver and muscle glycogen repletion has been observed with nutritional interventions, and based on the time course of glycogen repletion where the opportunity for most intervention is within the first 5 h [7, 27]. Therefore, if ketone esters can alter glycogen repletion over a 4-hour recovery period, this would enable the proof-of-concept to test whether this has potential to translate into endurance capacity. Further work is needed to establish this proof-of-concept and then the translation over other timeframes. Finally, it should also be noted that the current study design resulted in elevated beta-hydroxybutyrate concentrations at the onset of the second running bout and therefore any effects (or lack thereof) on performance are due to the combination of elevated beta-hydroxybutyrate concentrations in recovery and during subsequent exercise.

In conclusion, ingestion of ketone esters in recovery from exercise lower glycemia and increase ingested sucrose retention. These changes in metabolism may benefit subsequent endurance performance (and/or cardiometabolic health) under specific conditions, but in the present study we found no evidence that subsequent endurance exercise capacity was affected, possibly due to the fatigue mechanisms relating to factors other than maintenance of euglycemia within the scenario tested, and/or gastrointestinal symptoms counteracting metabolic effects. The current data provide a rationale to further explore the effects of ketone esters in recovery from exercise on liver and muscle glycogen resynthesis and subsequent endurance performance.

## Supplementary Information

Below is the link to the electronic supplementary material.

Supplementary Material 1
