# Supplementary material for: Ketone ester ingestion increases exogenous carbohydrate storage and lowers glycemia during post-exercise recovery: a randomised crossover trial
Source: Eur J Nutr. 2025 Aug 12;64(6):253. doi: 10.1007/s00394-025-03784-w (PMC12343750; doi:10.1007/s00394-025-03784-w)
Supplement: Supplementary file 1 — Supplementary Material 1 [file 394_2025_3784_MOESM1_ESM.docx]

| **Participant number** | **Laboratory visit 1** | **Laboratory visit 2** |
| --- | --- | --- |
| 1 | KET | PLA |
| 2 | PLA | KET |
| 3 | PLA | KET |
| 4 | PLA | KET |
| 5 | PLA | KET |
| 6 | KET | PLA |
| 7 | KET | PLA |
| 8 | PLA | KET |
| 9 | KET | PLA |
| 10 | KET | PLA |
| 11 | KET | PLA |
| 12 | KET | PLA |
| 13 | PLA | KET |

**Table S1. Randomised order of laboratory visits**

KET, ketone ester; PLA, placebo.
